# Genome-wide analyses of long non-coding RNA expression profiles and functional network analysis in esophageal squamous cell carcinoma

**DOI:** 10.1038/s41598-019-45493-5

**Published:** 2019-06-24

**Authors:** Junliang Ma, Yuhang Xiao, Bo Tian, Shaolin Chen, Baihua Zhang, Jie Wu, Zhining Wu, Xu Li, Jinming Tang, Desong Yang, Yong Zhou, Hui Wang, Min Su, Wenxiang Wang

**Affiliations:** 1 Department of the 2nd Department of Thoracic Surgery, Hunan Cancer Hospital and The Affiliated Cancer Hospital of Xiangya School of Medicine, Central South University, Changsha, Hunan 410013 P.R. China; 2 Hunan University of Medicine, Huaihua, Hunan 418000 P.R. China; 3 Hunan Key Laboratory of Translational Radiation Oncology, Hunan Cancer Hospital and The Affiliated Cancer Hospital of Xiangya School of Medicine, Central South University, Changsha, Hunan 410013 P.R. China; 4 Department of Pharmacy, Xiangya Hospital of Xiangya School of Medicine, Central South University, Changsha, Hunan 410001 P.R. China

**Keywords:** Cancer prevention, Tumour biomarkers, Diseases, Gene expression

## Abstract

Esophageal cancer (EC) is a serious malignancy and that is the fifth leading cause of cancer-related death worldwide. Esophageal squamous cell carcinoma (ESCC) is the main subtype of EC in China. In recent years, long non-coding RNAs (lncRNAs) have demonstrated to be novel tumor-associated regulatory factors. However, the functions and mechanisms of lncRNAs in ESCC have not been fully understood. In this study, we attempted to construct Genome-wide expression profiles of lncRNAs and their potential functions in ESCC. By using microarray, we found a total of 2,366 lncRNAs (1,032 upregulated and 1,334 downregulated) and 3,052 mRNAs (1,477 upregulated and 1,575 downregulated) were differentially expressed between the paired five ESCC tumor tissues and adjacent normal esophageal tissues (fold change, FC ≥2.0 or ≤0.5, p ≤ 0.05). Eight lncRNAs were detected by qRT-PCR to verify the results of the microarray, and the clinicopathological parameters were analyzed in 53 patients with ESCC. GO analysis and KEGG pathway analysis showed that the main biological functions of these abnormal lncRNAs were related to immune response, extracellular vesicular exosome, and protein binding. At the same time, the cis and trans models were used to analyze the potential synergistic regulatory relationship between lncRNAs and their potential target genes. Related genes were the processes that affect cell growth, differentiation, and migration. Then we mapped the lncRNAs-mRNAs co-expression pattern by calculating the PCCs of each lncRNA and mRNA expression value. Furthermore, we investigated the function and potential mechanism of a novel highly expressed lncRNA, lnc-KIAA1244-2, and found that its expression is associated with tumor size, N classification and clinical stage. Knockdown of lnc-KIAA1244-2 inhibited the cell proliferation and inhibited the TNFAIP3 expression in Eca-109 cells. Taken together, the expression patterns of lncRNAs and mRNAs in ESCC tumor tissues are different from those in normal adjacent tissues, and some abnormal expressed lncRNAs may play important roles in the development and progression of ESCC. Lnc-KIAA1244-2 could promote the cell proliferation of ESCC cells and might be a potent therapeutic target for ESCC.

## Introduction

Esophageal cancer (EC) is one of the most common fatal tumors, whose incidence is eighth and the mortality is fifth of all malignant tumors in the world^1^. There are two major subtypes of EC, esophageal squamous cell carcinoma (ESCC) and esophageal adenocarcinoma (EA)^2,3^. In China, ESCC accounts for more than 90%^4^ of the total cases and the 5-year survival rate of the advanced stage is less than 30%^5^. So far, the origin of ESCC carcinogenesis has not been identified well^6^. Therefore, it is required to identify specific and reliable biomarkers involved in development and progression of ESCC to predict the clinical outcome of the tumor.

In recent years, non-coding RNA (ncRNA) has been demonstrated to be involved in the progression of disease, including cancer^7,8^. Increasing studies have focused on long non-coding RNAs (lncRNAs), which are greater than 200 nt and are unable to be translated into proteins^9–11^. Studies have shown that lncRNAs are involved in a variety of biological processes and play important roles in tumorigenesis and tumor progression^12–15^.

There is increasing evidence that lncRNAs may act as a tumor suppressor or promoter in various tumors and may be a new class of tumor biomarkers and therapeutic targets^12^. For instance, lncRNA MIR31HG is upregulated in ESCC tumor tissues and plasma, which is related to TNM stage and lymph node metastasis. Knockdown of MIR31HG can inhibit the proliferation, migration, invasion, and inhibited the expression of Furin and MMP1 in EC9706 and EC1 cells. It can be used as biomarkers for diagnosis or prediction of ESCC^16^. The discovery of lncRNAs provides a new strategy to understand the mechanism of tumor occurrence and development.

In the present study, we screened the differential expression profiles of lncRNAs in ESCC tissues by microarray and analyzed differentially expressed lncRNAs function and potential related acting molecules to further understand the role of lncRNAs in the occurrence and development of ESCC. In addition, we showed that lnc-KIAA1244-2 was upregulated in ESCC and could promote the proliferation of ESCC. Lnc-KIAA1244-2 might be a potent therapeutic target for ESCC.

## Results

### Expression profiles of lncRNAs in ESCC

**Figure 1:**
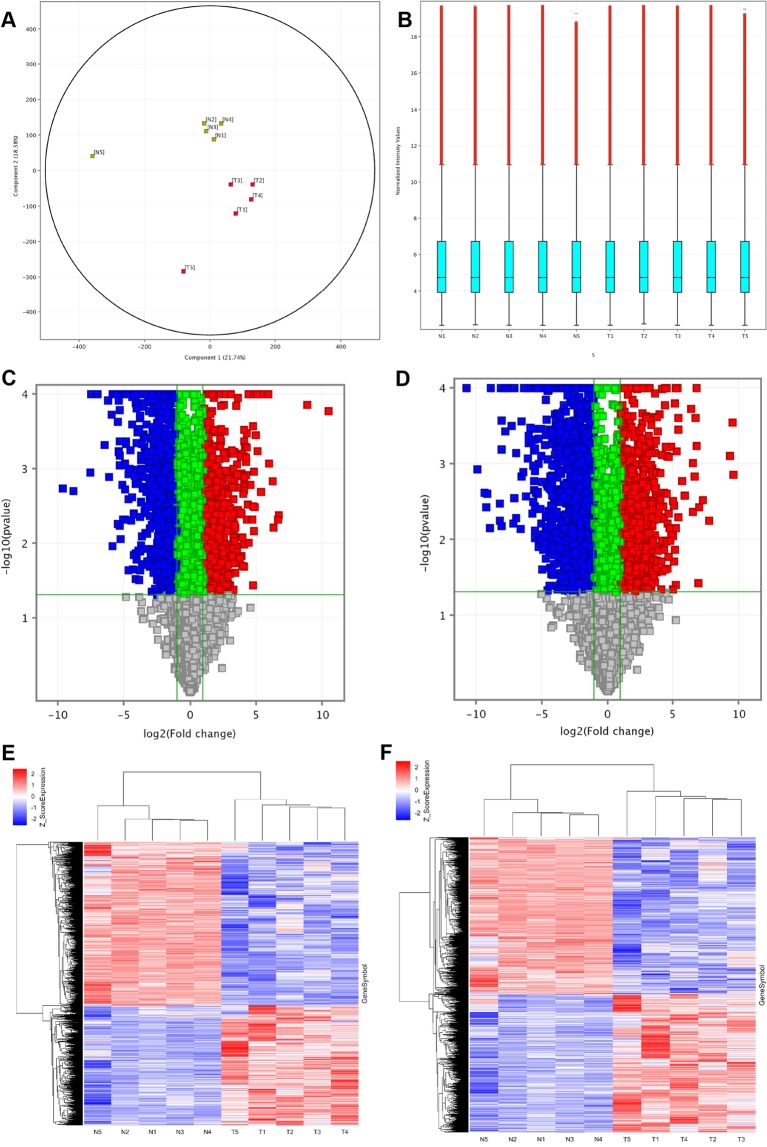
LncRNAs and mRNAs expression profiles in ESCC. (**A**) Principal Components Analysis. The red boxes represent the ESCC tumor tissues. The yellow boxes represent adjacent normal esophagus tissues. (**B**) Validation of differential lncRNA expression and function. The box plot is a convenient method to quickly compare the distribution of lncRNAs. After normalization, the distributions of log 2 ratios among the tested samples are almost similar. (**C**) Volcano Plot of the differentially expressed lncRNAs and (**D**) mRNAs in ESCC tumor tissues and adjacent normal esophagus tissues. The red points in the plot represent differentially expressed lncRNAs with statistical significance. (**E**) Hierarchical Clustering shows a distinguishable lncRNA expression profile and (**F**) mRNA expression profile. T, ESCC tumor tissue. N, adjacent normal esophagus tissues.

To construct expression profiles of lncRNAs in ESCC, we used microarray to detect the expression of lncRNAs, as well as mRNAs in five pairs of ESCC tumor tissues and adjacent normal tissues. The expression of lncRNAs and mRNAs in ESCC tumor tissues was significantly different from that of the adjacent normal esophageal tissues. Principal component analysis (Fig. 1A) was applied to investigate the distribution of samples and verify the rationality of the experimental design. The samples in the same group were concentrated in two-dimensional space, indicating that these genes were selected and the biological repeats were good. The box plot was used to quickly compare the distribution of lncRNAs, and after normalization, the distributions of log 2 ratios among the tested samples were almost similar (Fig. 1B). Volcano plots were used to outline the misalignment of lncRNAs and mRNAs in datasets (Fig. 1C,D). The lncRNAs expression profile (Fig. 1E) and mRNAs expression profile (Fig. 1F) were established and clustered using hierarchical cluster analysis. Based on the microarray data, 3,052 lncRNAs and 2,366 mRNAs were identified to be differentially expression (FC ≥2.0 or ≤0.5, p ≤ 0.05). Among those, 1,032 and 1,334 lncRNAs were upregulated and downregulated, respectively; 1,477 and 1,575 mRNAs were upregulated and downregulated, respectively. The top 30 upregulated and downregulated lncRNAs and mRNAs are listed in Table 1. The most upregulated lncRNA and mRNA were lnc-MMP10-3 (absolute FC = 1469.9) and SPP1 (absolute FC = 774.8), respectively. The most downregulated lncRNA and mRNA were lnc-FLG2-2 (absolute FC = 817.0) and CRISP3 (absolute FC = 1701.3), respectively.

**Table 1:** The top 30 up- and downregulated lncRNAs and mRNA in ESCC tumor tissues compared with adjacent normal esophageal tissues.

Upregulated lncRNAs		Downregulated lncRNAs		Upregulated mRNAs		Downregulated mRNAs	
GeneSymbol	Fold change	GeneSymbol	Fold change	Fold change	Fold change	GeneSymbol	Fold change
lnc-MMP10-3	1469.95	lnc-FLG2-1	816.99	SPP1	774.77	CRISP3	1701.30
lnc-MMP10-2	479.94	lnc-FLG2-2	459.18	MMP1	744.55	MAL	966.54
lnc-MMP3-1	104.95	lnc-KRT79-1	196.39	CST1	624.71	CRNN	671.34
lnc-PKD2-2	78.58	lnc-MTX2-6	180.21	MMP13	210.90	MUC21	611.75
lnc-CXCL6-2	76.84	lnc-KRT79-2	131.82	MMP12	149.47	FLG	590.34
lnc-LIPH-4	62.33	lnc-ANXA8L2-1	91.04	POPDC3	125.62	CLCA4	491.91
lnc-GULP1-4	61.28	lnc-LIPI-5	76.56	CALB1	121.32	UPK1A	470.63
lnc-GULP1-2	61.26	lnc-KRT36-1	72.72	COL1A1	109.90	SPINK7	301.17
lnc-SLCO1B3-1	41.18	RP11-641D5.2	62.08	POSTN	109.26	TMPRSS11B	300.16
lnc-DHX32-3	38.94	lnc-OCIAD1-1	59.97	SFRP4	105.51	IL36A	282.00
NONHSAG033144	34.13	lnc-EPGN-1	59.51	HOXD11	99.80	KRT4	271.47
lnc-XRCC4-6	34.01	lnc-LIPI-4	57.32	CASC9	89.95	LOC101927354	268.85
lnc-COL1A1-5	33.26	lnc-MC5R-11	57.23	IL8	89.55	CRCT1	264.57
lnc-DHX32-4	30.83	C21orf15	50.62	ADAM12	82.63	DYNAP	222.29
lnc-CXCL6-3	29.93	lnc-STXBP5-6	45.76	CXCL10	57.90	EPGN	220.81
lnc-LAMC1-1	27.78	lnc-TARS2-1	45.37	PLA2G7	51.47	KRT78	198.32
lnc-CASD1-2	27.75	lnc-AC073416.2-11	43.18	LAMC2	42.92	ENDOU	156.99
lnc-TRPC4-2	27.72	lnc-FAM25B-1	42.63	COL5A1	42.56	KRT13	148.28
lnc-ELMO1-2	27.58	OTTHUMG00000133684	42.61	OLFML2B	41.55	HPGD	139.61
lnc-FADD-2	27.12	lnc-KIF7-3	42.18	COL1A2	39.82	ALOX12	136.49
lnc-SAMD14-5	26.95	lnc-OCIAD1-2	40.82	MIR3606	38.79	FAM25A	125.79
lnc-GLI3-4	25.49	lnc-AC073416.2-11	40.81	NELL2	37.43	SCEL	123.57
LINC01296	23.48	lnc-PHC1-2	40.75	LOC101060271	36.96	CWH43	99.16
lnc-GULP1-3	21.70	lnc-ARID5B-1	40.39	THY1	36.52	PPP1R3C	93.55
lnc-ATP2B3-3	21.57	lnc-VSIG10L-2	38.78	AMTN	36.05	SH3BGRL2	92.50
lnc-NMI-2	20.30	lnc-AFAP1L1-3	37.14	ANO1	34.68	A2ML1	85.55
lnc-POTEM-2	19.78	lnc-SPRR1A-2	35.98	IFI6	34.25	EMP1	82.51
lnc-SMAD5-7	19.61	lnc-KRT15-2	34.92	RP1-27K12.2	33.41	FAM3B	73.05
lnc-GCG-1	19.51	HCG22	33.14	MFAP2	32.88	PADI1	67.12
lnc-POTEG-4	18.83	lnc-SPINK7-1	31.87	SLCO1B3	32.31	SERPINB2	66.37

### Validation of differential lncRNA expression

**Figure 2:**
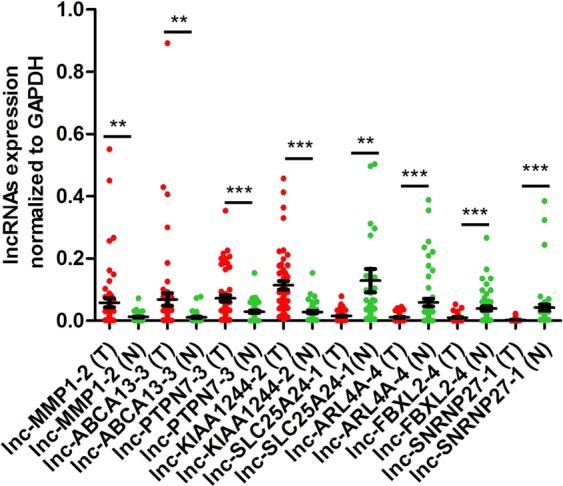
QRT-PCR analysis of the selected 8 lncRNAs. **p < 0.01; ***p < 0.001; T, ESCC tumor tissue; N, adjacent normal esophagus tissues.

To verify the reliability of the microarray, we randomly selected 8 notably differentially expressed lncRNAs, including upregulated lnc-MMP1–2 (absolute FC = 10.00), lnc-ABCA12-3 (absolute FC = 9.26), lnc-PTPN7-3 (absolute FC = 6.65), lnc-KIAA1244-2 (absolute FC = 6.24), and downregulated lnc-SLC25A24-1 (absolute FC = 12.80), lnc-ARL4A-4 (absolute FC = 12.12), lnc-FBXL2–4 (absolute FC = 6.69), lnc-SNRNP27-1 (absolute FC = 7.05), and used qRT-PCR analysis to verify the microarray results in 53 pairs of ESCC tumor tissues and adjacent normal tissues. The results indicated that these lncRNAs were upregulated or downregulated in the ESCC tumor group (p < 0.05), consistent with the results of microarray (Fig. 2).

### Potential function recognition of the differential expression lncRNAs in ESCC

**Figure 3:**
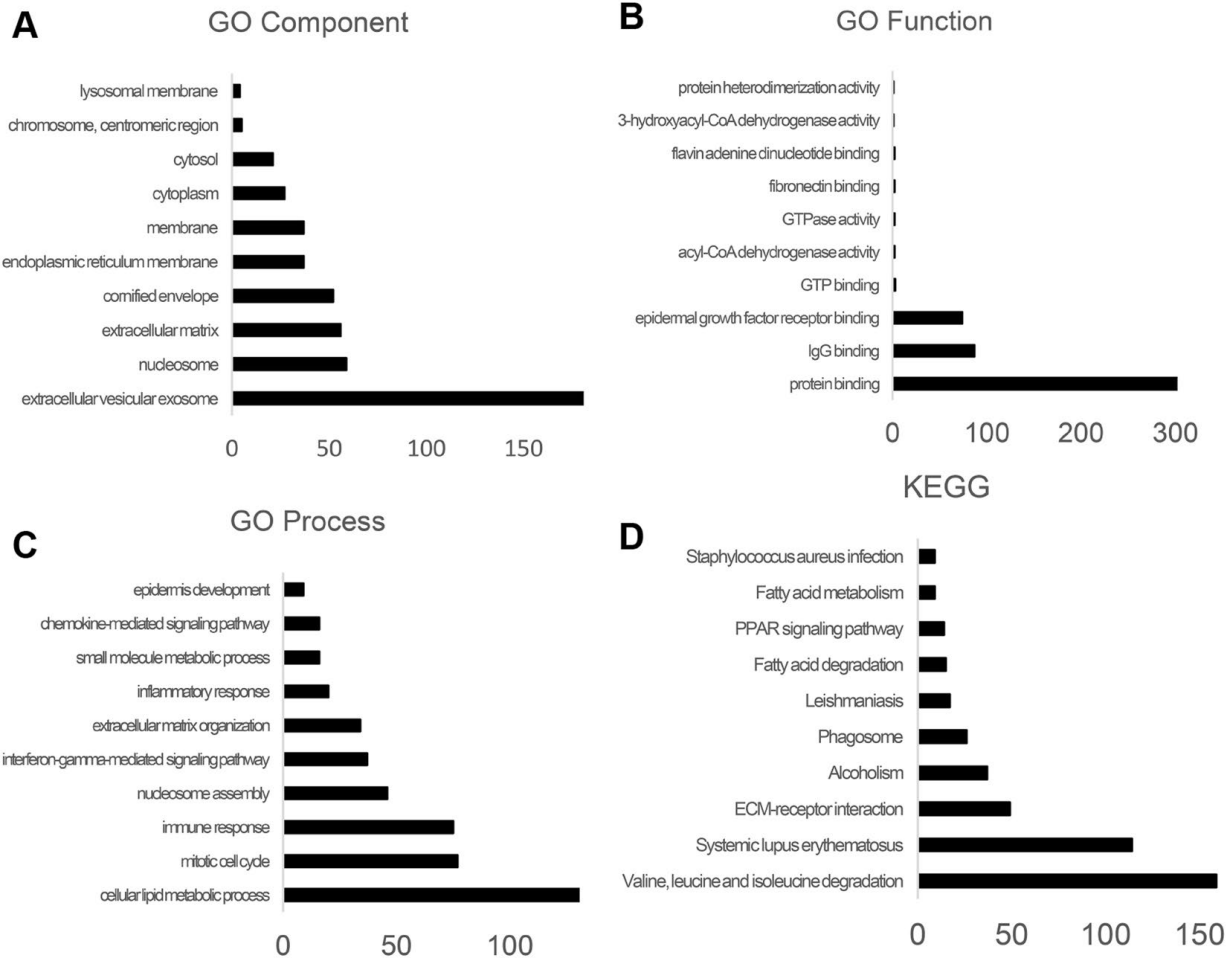
Go and KEGG Pathway analysis of lncRNAs co-expressed genes. The top 10 enriched terms were calculated as −log10 (p-value).

We further analyzed the differential expression of lncRNAs between ESCC tumor tissues and normal tissues by GO analysis and KEGG pathway analysis. The top 500 terms in the GO terms list were highly enriched for immune response, mitotic cell cycle, cellular lipid metabolic process (ontology: biological process), extracellular vesicular exosome, nucleosome, extracellular matrix (ontology: cellular component) and protein binding, and immunoglobulin G binding (ontology: molecular function). The top 500 terms in the KEGG pathway were associated with valine, leucine and isoleucine degradation, systemic lupus erythematosus, extracellular matrix receptor interaction, alcoholism, and phagosome. The GO and KEGG pathway analyses are shown in Fig. 3A–D.

To explore the relationship between differentially expressed lncRNA and target mRNA in ESCC for each differentiated lncRNA, we calculated the enrichment of functional terms of co-expressed genes in top 400 differentially expressed lncRNAs. The highest up-regulated lncRNA was lnc-MMP10-3 (absolute FC = 1469.95) among ESCC tissues versus paired non-carcinoma tissues and a total of 809 genes (e.g. S100A16, OLFML2B, and CAST) were related to lnc-MMP10-3 as the standard of absolute PCCs value > 0.8. The top 30 co-expressed genes of lnc-MMP10-3 are listed in Table S2 (see Supplementary). Furthermore, GO and KEGG pathways were applied to annotate the lnc-MMP10-3 co-expressed mRNA function. The top 30 reliably predicted terms of GO analysis are listed in Table S3 (see Supplementary). It shows that significantly enriched GO terms were involved in fatty-acyl-CoA binding, G1/S transition of mitotic cell cycle, Golgi membrane, MAPK cascade, and transfer RNA-intron endonuclease activity. Moreover, KEGG pathways analysis results, including fatty acid elongation, primary bile acid biosynthesis, valine, leucine, and isoleucine degradation, glutathione metabolism, and amino sugar and nucleotide sugar metabolism, are listed in Table S4 (see Supplementary).

### LncRNAs target prediction

**Figure 4:**
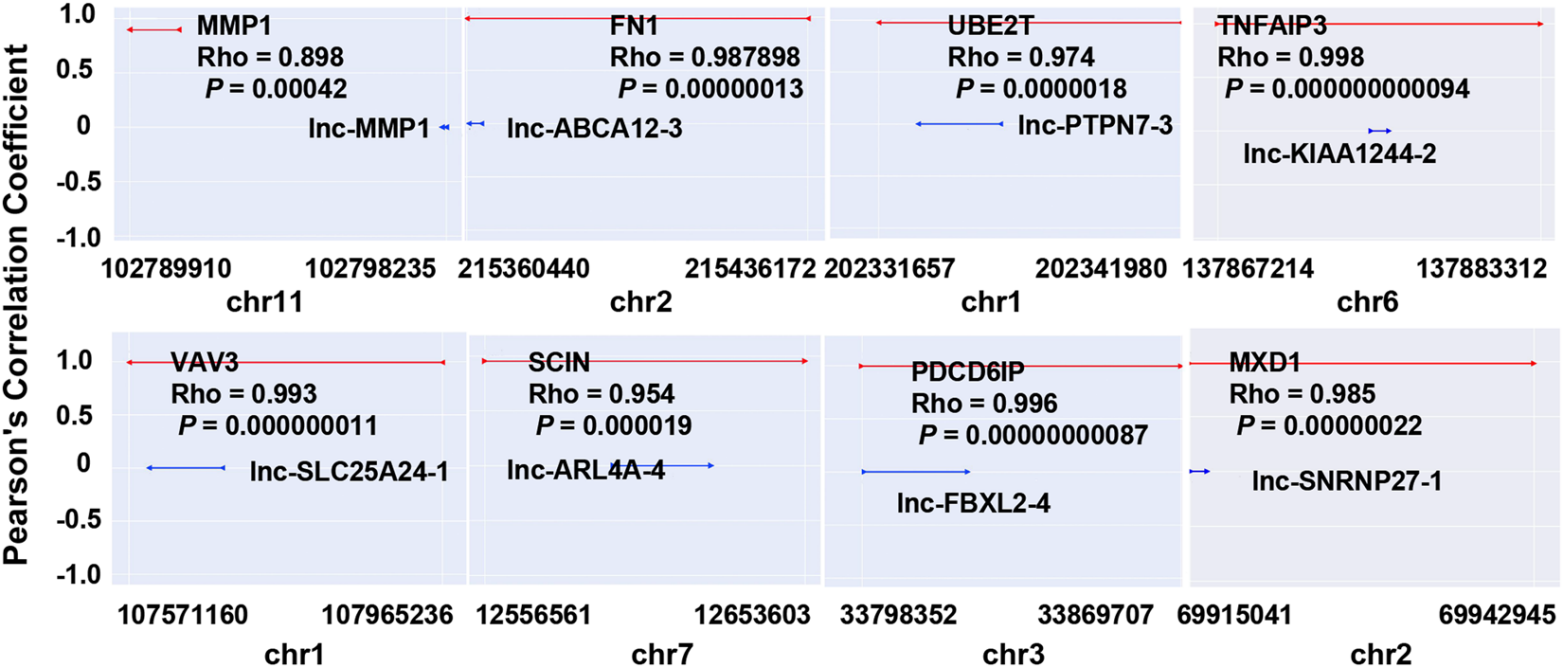
Cis-regulation genes of lncRNAs in the chromosome. The Y-axis represents correlation coefficient of lncRNA and potential “cis” genes. The blue line represents the genome width of lncRNA and red line represents the position of potential “cis” genes.

To explore the roles of lncRNAs in ESCC, we used co-expression network analysis to predict cis- or trans-regulatory genes for differentially expressed lncRNAs. The cis relationship of 8 lncRNAs (lnc-MMP1–2, lnc-ABCA12-3, lnc-PTPN7-3, lnc-KIAA1244-2, lnc-SLC25A24-1, lnc-ARL4A-4, lnc-FBXL2–4 and lnc-SNRNP27-1) is shown in Fig. 4.

**Figure 5:**
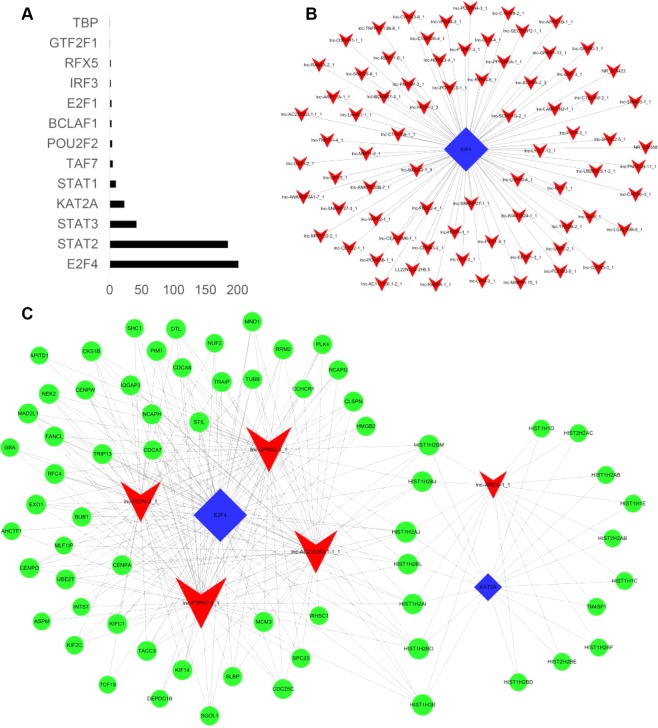
(**A**) Frequency distribution of lncRNAs enrichment on TFs. The X-axis is frequency distribution and Y-axis is the TFs name. (**B**) LncRNAs-TFs analysis. Network of the top 100 lines regulating relations of lncRNAs-TFs (consist of 75 lncRNAs and 1 TF). (**C**) TFs-lncRNAs-target genes. Network of the top 10 lines regulating relations of TFs-lncRNAs-target genes (consist of 5 lncRNAs, 2 TFs and 87 target genes).

In addition, the enrichment of differentially expressed lncRNAs co-expressed coding genes in TFs indicated that a total of 500 lncRNAs were regulated by 13 TFs (Fig. 5A) and may be mostly regulated by the 5 TFs (E2F4, STAT2, STAT3, KAT2A, STAT1) (Fig. 5B). These TFs reflect the overall functional distribution of the expressed lncRNAs. The top 100 regulating relations based on P value to draw binary-relation network diagrams by using Cytoscape software. In Fig. 5B, 75 lncRNAs and the transcription factor E2F4 were involved. Considering lncRNA-TF was derived from the enrichment of various genes, we drew ternary-relation network diagrams on account of the top 10 regulating relations (Fig. 5C). It includes 2 upregulated lncRNAs (lnc-GPR50-3 and lnc-PTPN7-3) and 3 downregulated lncRNAs (lnc-AREG-1, lnc-PCP4-2, and lnc-AC233263.1), 2 TFs (E2F4 and KAT2A), and 87 target genes in this map.

### Relationship between lncRNAs and clinicopathological parameters of ESCC

To investigate the clinicopathological significance of lncRNAs (lnc-MMP1-2, lnc-ABCA12-3, lnc-PTPN7-3, lnc-KIAA1244-2, lnc-SLC25A24-1, lnc-ARL4A-4, lnc-FBXL2–4 and lnc-SNRNP27-1) expressions in ESCC patients, the patients were classified by sex, age, smoking index, drinking index, differentiation, TNM stages, and clinical stage based on the median expression level of analyzed lncRNAs. As shown in Table 2, We found that lnc-MMP1–2 expression was negative associated with age (p = 0.013) and pathologic differentiation (p = 0.031), lnc-ABCA12-3 expression was positively associated with N classification (P = 0.002) and clinical stage (p = 0.006), lnc-KIAA1244-2 expression was positively associated with tumor size (p < 0.001), N classification (p = 0.013) and clinical stage (p = 0.006), lnc-SLC25A24-1 was negative associated with age (p = 0.002), lnc-SNRNP27-1 was negative associated with tumor size (p = 0.042) and T classification (p = 0.035).

**Table 2:** The association between lncRNA expression and ESCC patient’s clinicopathological.

Characteristics features	lnc-MMP1-2		*P* ^a^	lnc-ABCA12-3		*p* ^a^	lnc-PTPN7-3		*P* ^a^	lnc-KIAA1244-2		*P* ^a^	lnc-SLC25A24-1		*P* ^a^	lnc-ARL4A-4		*P* ^a^	lnc-FBXL2-4		*P* ^a^	lnc-SNRNP27-1		*P* ^a^
	Low	High		Low	High		Low	High		Low	High		Low	High		Low	High		Low	High		Low	High	
Age (years)			0.013			0.786			0.414			0.276			0.002			0.173			0.056			1.000
<60	8	18		12	14		11	15		15	11		7	19		10	16		9	17		13	13	
≥60	18	9		14	13		15	12		11	16		19	8		16	11		17	10		13	14	
Gender			1.000			1.000			1.000						1.000			0.236			1.000			1.000
Male	25	26		25	26		25	26		24	27	0.236	25	26		24	27		25	26		25	26	
Female	1	1		1	1		1	1		2	0		1	1		2	0		1	1		1	1	
Smoking Index			0.704			0.704			0.420			1.000			0.250			0.050			1.000			1.000
Yes	22	24		22	24		24	22		23	23		21	25		20	26		23	23		23	23	
No	4	3		4	3		2	5		3	4		5	2		6	1		3	4		3	4	
Drinking Index			0.175			0.728			0.728			0.501			0.501			0.175			0.728			0.728
Yes	19	24		22	21		22	21		20	23		20	23		19	24		22	21		22	21	
No	7	3		4	6		4	6		6	4		6	4		7	3		4	6		4	6	
Tumor location			0.755			0.724			0.724			0.091			0.203			0.252			0.464			0.464
Upper	3	2		3	2		3	2		1	4		3	2		4	1		3	2		3	2	
Middle	12	15		14	13		14	13		17	10		10	17		11	16		11	16		11	16	
lower	11	10		9	12		9	12		8	13		13	8		11	10		12	9		12	9	
Differentiation			0.031			0.509			0.890			0.405			0.076			0.209			0.260			0.166
Well	4	5		4	5		4	5		5	4		2	7		2	7		3	6		4	5	
moderate	21	14		19	16		18	17		15	20		21	14		19	16		20	15		15	20	
Poor	1	8		3	6		4	5		6	3		3	6		5	4		3	6		7	2	
Tumor size (cm)			0.773			1.000			0.387			<0.001			0.387			0.773			0.773			0.042
≤3	8	10		9	9		7	11		16	2		7	11		8	10		8	10		5	13	
>3	18	17		17	18		19	16		10	25		19	16		18	17		18	17		21	14	
T-classifcation			0.559			1.000			0.766			0.077			0.559			1.000			0.241			0.035
T1-2	9	7		8	8		7	9		11	5		9	7		8	8		10	6		4	12	
T3-4	17	20		18	19		19	18		15	22		17	20		18	19		16	21		22	15	
N-classifcation			1.000			0.002			0.786			0.013			0.586			1.000			0.586			0.586
N0	12	13		18	7		13	12		17	8		11	14		12	13		11	14		11	14	
N1-3	14	14		8	20		13	15		9	19		15	13		14	14		15	13		15	13	
Clinical stage			0.586			0.006			0.586			0.006			1.000			0.586			0.586			0.173
I–II	15	13		19	9		15	13		19	9		14	14		15	13		15	13		11	17	
III	11	14		7	18		11	14		7	18		12	13		11	14		11	14		15	10	

*p*, p-value; ^a^Chi-squared or Fisher’s exact tests.

### Knockdown of lnc-KIAA1244-2 inhibited the cell proliferation and inhibited the TNFAIP3 expression in Eca-109 cells

**Figure 6:**
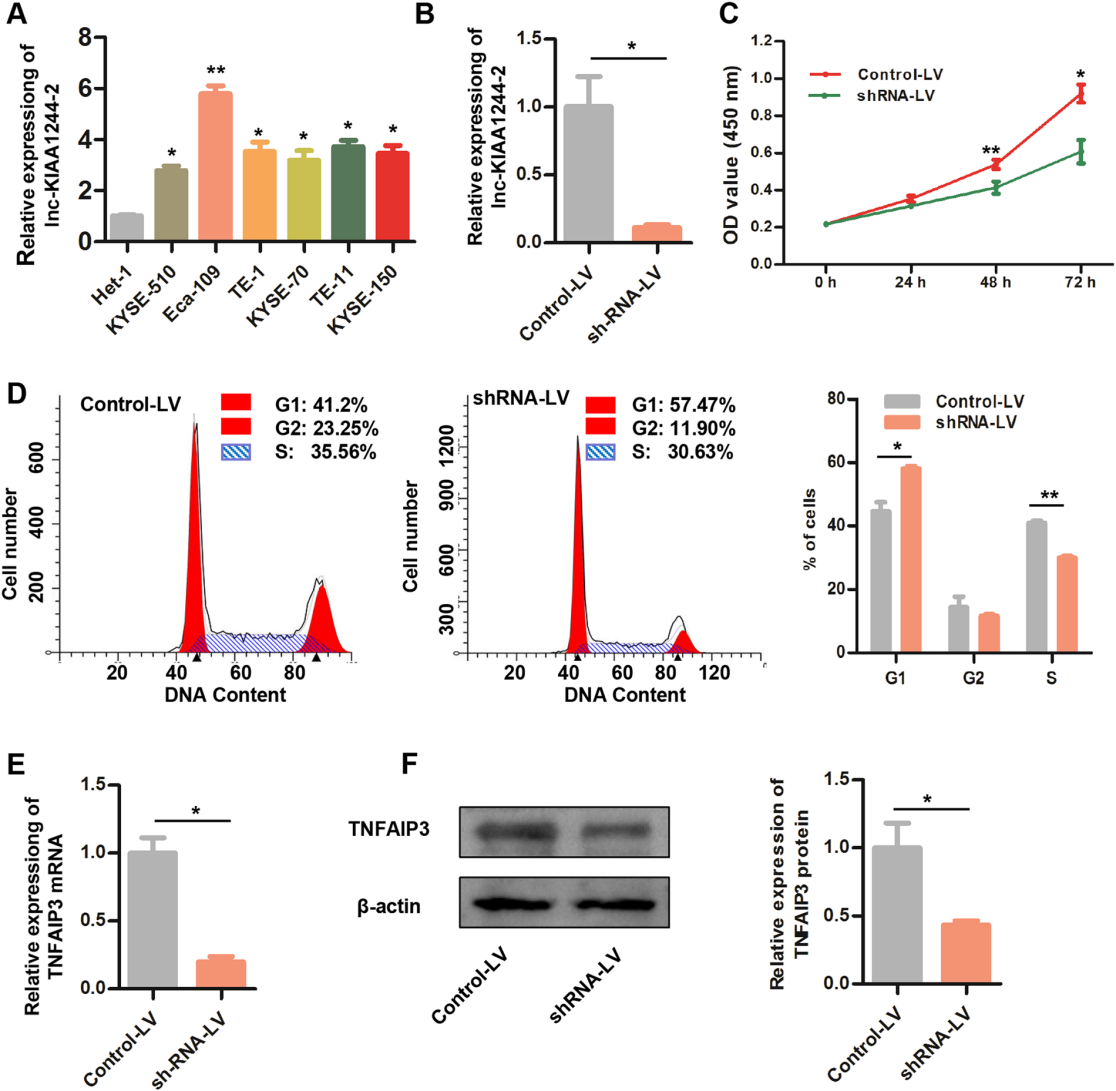
Lnc-KIAA1244-2 knockdown analysis. (**A**) The expressions of lnc-KIAA1244-2 in normal human esophageal epithelial-1 cell (Het-1A) and ESCC cell lines KYSE-510, Eca-109, TE-1, KYSE-70, TE-11, and KYSE-150 were detected by qRT-PCR. (**B**) Lentivirus-Mediated Small Hairpin RNA decreased lnc-KIAA1244-2 expression in ESCC cell line Eca-109. (**C**) CCK-8 assay. Knockdown of lnc-KIAA1244-2 suppressed the proliferation of Eca-109 cells. (**D**) Cell cycle analysis. Knockdown of lnc-KIAA1244-2 significantly increased the percentage of Eca-109 cells in the G0/G1 phase, and significantly decreased the S-phase fractions. (**E**) QRT-PCR assay. Knockdown of lnc-KIAA1244-2 inhibited TNFAIP3 mRNA expression in Eca-109 cells. (**F**) Western blot assay. Knockdown of lnc-KIAA1244-2 inhibited TNFAIP3 protein expression in Eca-109 cells. The full-length blots/gels are presented in Supplementary Fig. S1. *p < 0.05; **p < 0.01, as compared with the controls-LV cells. Control-LV, infected with negative lentivirus; shRNA-LV, infected with lenti-shRNA lnc-KIAA1244-2.

To further investigate the functions and mechanisms of lncRNAs in ESCC, one of the upregulated lncRNAs, lnc-KIAA1244-2, was identified for further investigation in ESCC cells. As shown in Fig. 6A, the expression levels of lnc-KIAA1244-2 in the ESCC cell lines were higher than that in the normal esophageal epithelial cell line Het-1 A and was prominently upregulated in the Eca-109 cell lines.

Furthermore, short hairpin RNA (shRNA) mediated by a lentiviral vector (LV) was used to knockdown lnc-KIAA1244-2 in Eca-109 cells. As shown in Fig. 6B, the expression level of lnc-KIAA1244-2 was significantly downregulated in the shRNA-LV groups compared with the expression level in the control-LV group.

The CCK-8 assays and cell cycle assays indicated that the knockdown of lnc-KIAA1244-2 observably inhibited the proliferation of Eca-109 cells (Fig. 6C). Cell cycle analysis demonstrated that lnc-KIAA1244-2 knockdown led to cell cycle arrest at G0/G1 phase (Fig. 6D). These results indicated that lnc-KIAA1244-2 promoted the proliferation of ESCC cells. In addition, the mRNA level and protein level of TNFAIP3 were detected in lnc-KIAA1244-2 knockdown Eca-109 cells, since TNFAIP3 was predicted to be the target of lnc-KIAA1244-2. The results in Fig. 6E,F showed that both the TNFAIP3 mRNA and protein levels were downregulated after lnc-KIAA1244-2 knockdown in Eca-109 cells, indicating lnc-KIAA1244-2 might regulate ESCC cell proliferation via mediating TNFAIP3.

## Discussion

In the past 50 years, researchers have concentrated on the role of mRNA in disease processes. However, the Human Genome Project found that fewer than 2% of the genomes encode proteins, more than 98% of the genomes transcribe ncRNAs, and most ncRNAs were considered to be transcribed to lncRNAs^17^. In view of the important functional roles revealed by small ncRNAs, these lncRNAs have received increasing attention in recent years in their possible functions^6^. Some ESCC-related lncRNAs have been identified, such as lncRNA SBF2-AS1, AK001796, and MIR31HG, and can be used as biomarkers for the diagnosis or prediction of ESCC^16,18,19^. However, the amount of identified lncRNAs is only a small fraction, and the function of a large number of lncRNAs requires further investigation.

In this study, we screened the genome-wide expression profiles of lncRNAs and mRNAs in ESCC and found that the expression of lncRNAs and mRNAs in ESCC tumor tissues were significantly different from that of the adjacent normal esophageal tissues. It indicates that lncRNAs are abnormally expressed in ESCC. We randomly selected eight dysregulated lncRNAs (lnc-MMP1-2, lnc-ABCA12-3, lnc-PTPN7-3, lnc-KIAA1244-2, lnc-SLC25A24-1, lnc-ARL4A-4, lnc-FBXL2-4 and lnc-SNRNP27-1) to verify microarray data by qRT-PCR. The results were consistent with the results of the microarray data, indicating that the microarray data are credible.

Then we analyzed the target genes of the top 400 differentially expressed lncRNAs based on co-expressed analysis and predicted the function of the top 500 lncRNAs using GO analysis and KEGG pathway. Most of these potential cis-regulatory target genes, including cell growth, differentiation, and migration, are associated with tumorigenesis and tumor progression. The lncRNA expression profiles overlapping our current study and previous studies confirmed the reliability of this study. For example, SOX2OT is highly expressed in ESCC tumor tissues^20,21^. In our data, SOX2OT expression was upregulated with an average expression FC of 2.0. Our data also suggested that cis-regulated target genes were involved in the initiation and progression of ESCC. For instance, FN1 is highly expressed in a variety of tumor tissues including ESCC, which can promote tumor metastasis and invasion^22–24^. In our data, FN1 was found to be highly expressed in ESCC tissues (FC = 11.5).

LncRNAs can interact directly with gene promoters and TFs. These lncRNAs enhance and regulate the activity of promoters by recruiting protein factors^25^. LncRNAs control the transcription process through their interaction with primary coding transcripts. The differential expression lncRNAs may be mostly regulated by the 5 TFs (E2F4, STAT2, STAT3, KAT2A, STAT1). E2F4 is a cyclical protein and a transcription factor with transcriptional inhibition. STAT1 and STAT2 are associated with the regulation of cell apoptosis. STAT3 as an oncogene is associated with the regulation of cell proliferation^26^. KAT2A is an acetyltransferase that alters the structure of DNA by transferring acetyl groups. At the same time, some studies have shown that KAT2A plays an important role in regulating the function of stem cells, and can promote the normal development of embryos^27,28^, thereby enhancing the transcriptional level of specific DNA and selectively producing various traits.

In addition, we investigated the association between expressions of the validated eight lncRNAs and clinicopathological parameters in ESCC. The results showed that multiple lncRNAs were associated with clinicopathological parameters. We found that lnc-KIAA1244-2 expression was positively associated with tumor size, N classification and clinical stage. Thus, the function and mechanism of lnc-KIAA1244-2 were further studied. Lnc-KIAA1244-2 is 234 nucleotides in length and is located on chromosome 6q23.3. An important role of lncRNAs is to regulate the expression of adjacent protein-coding genes through cis regulatory model^13^. The results of cis-regulation showed that the genes encoding TNFAIP3 and lnc-KIAA1244-2 are located at the same locus. It was previously reported that TNFAIP3 is involved in proliferation, migration and invasion of EC cells^29^. By knockdown assays, we demonstrated that lnc-KIAA1244-2 could promote cell proliferation and regulate TNFAIP3 expression. Therefore, we speculate that lnc-KIAA1244-2 promotes the progression of ESCC by regulating TNFAIP3. Lnc-KIAA1244-2 might be a biomarker of diagnosis and prognosis and a therapy target of ESCC. However, functions and mechanisms of lnc-KIAA1244-2 need further study.

In conclusion, this study demonstrates that the expression patterns of lncRNAs and mRNAs in ESCC tumor tissues are different from those in normal adjacent tissues, and some abnormal expression of lncRNAs may play important roles in the development and progression of ESCC. The profiles we established in ESCC will provide a foundation for further study on the mechanism of differentially expressed lncRNA in ESCC and their clinical significance. These lncRNAs identified in this study are promising biomarkers for diagnosis, classification, prognosis, and therapeutic evaluation in patients with ESCC.

## Materials and Methods

### Samples

In this experiment, 53 pairs of ESCC tumor tissues and their adjacent normal esophageal tissues (2.0 cm from the edge of tumor tissue) were collected from June 2015 to January 2016 in the 2nd Department of Thoracic, Hunan Cancer Hospital/The Affiliated Cancer Hospital of Xiangya School of Medicine, Central South University. ESCC was diagnosed in all cases by pathology. Prior to esophagectomy, all patients (or family members of the patient) signed written consent and there was no distant metastasis in the imaging examination. None of the patients underwent radiotherapy or chemotherapy before surgery. After the specimens were removed, the ESCC tumor tissues and their adjacent normal tissues with a diameter of approximately 3.0 mm were harvested. Specimens were frozen in liquid nitrogen and then stored at −80°C in a refrigerator. Then, five pairs of tissues were selected for microarray analysis. A total of 53 pairs of tissues were selected for the clinicopathological analysis. All specimen collection and experimental methods were approved by the Institutional Ethics Committee of Hunan Cancer Hospital and performed in accordance with relevant regulations. Informed consent was obtained from all patients. The classification of tumor node metastasis (TNM) was based on the standard of the 7th edition of the TNM staging system.

### Generation of microarray data

Sample preparation and microarray hybridization were performed by OE Biotech Corporation, Shanghai, P.R. China. This experiment used an Affymetrix Human lncRNA array containing 63.542 lncRNAs probes and 27.134 mRNA probes. These probes were derived from six databases: lncRNAdb V1, Broad Institute (Human. Map lincRNAs), Ensemble, Refseq, NONCODE v4, and GeneBank. Total RNA was extracted by mirVanaTM RNA Isolation Kit (AM1561) and purified using QIAGENRNeasy® MiniKit. Total RNA integrity was measured by NanoDrop ND-2000 (Fisher Thermo Scientific) and its integrity was evaluated by Agilent Bioanalyzer 2100 (Agilent Technologies). The samples were labeled, hybridized, and washed depending on the standard protocols of the manufacturer. In short, the total RNA is transcribed into double-stranded complementary DNA (cDNA) and then synthesized into antisense RNA (aRNA). The second cycle of cDNAs were synthesized from aRNA, then fragmented and biotinylated followed by microarray hybridization. After washing and staining, the arrays were scanned by Affymetrix Scanner 3000 (Affymetrix).

### Quantitative real-time polymerase chain reaction validation

The results of the microarray were verified by quantitative real-time polymerase chain reaction (qRT-PCR). Total RNA was extracted by Trizol reagent (Ambion) and reverse transcribed by GoScript^TM^ reverse transcription system (GeneCopoeia). QRT-PCR was implemented using All-in-One^TM^ qPCR Mix (GeneCopoeia). GAPDH was used as an internal reference gene. Primers for qRT-PCR analysis of lncRNAs are listed in Table S1 (see Supplementary). The lncRNA expression levels between ESCC tumor tissues and adjacent normal tissues were compared using paired *t* test.

### Bioinformatics analysis

The extraction of raw data was based on Affymetrix Gene Chip Command Console (version 4, Affymetrix) software. RMA standardization was provided for gene and exon level analysis using Expression Console (version 1.3.1, Affymetrix) software. Gene expression analysis was performed using GeneSpring software (version 14.8, Agilent Technologies). Differentially expressed genes were identified by fold change (FC) and P values calculated with *t* tests. The threshold value for upregulated and downregulated genes was a FC ≥2.0 and p ≤ 0.05. Subsequently, Gene Ontology (GO: http://www.geneontology.org) analysis and Kyoto Encyclopedia of Genes and Genomes (KEGG: http://www.me.jp/kegg) analysis were used to determine the role of these differentially expressed lncRNAs. Finally, the differentially expressed lncRNAs and mRNAs in the samples were described by hierarchical clustering.

### Differential expression analysis

Differentially expressed lncRNAs and mRNAs were identified by paired *t* test (FC ≥2.0 or ≤0.5, p < 0.05 and FDR <0.05). The microarray data have been uploaded in NCBI Gene Expression Omnibus (GEO) and the GEO accession number is GSE120356 (http://www.ncbi.nlm.nih.gov/geo/query/acc.cgi?acc = GSE120356).

### Prediction of lncRNAs function

The positive correlation between lncRNAs and mRNAs was expressed as an absolute value of Pearson correlation ≥0.7 and p ≤ 0.05. The enrichment of co-expressed mRNAs was calculated by hypergeometric cumulative distribution function. Then we selected top 100 upregulated and downregulated lncRNAs and searched for co-expressed genes within the 100-kb window of each lncRNA (p ≤ 0.05). The genes on both sides of lncRNA were potential cis regulatory genes. A determination needed to be made as to which genes may be trans regulated by lncRNAs. The JEMBOSS software was used to determine which TFs might interact with lncRNAs. For each abnormal expression of lncRNA, coded genes were calculated and the significance of gene enrichment in each TF entry was calculated by hyper- geometric distribution test. Small P values indicate that the differential expression of the gene was enriched in the TF entry. We used Cytoscape software to draw TFs, mRNAs, and lncRNAs relational networks.

### Cell culture and culture conditions

The normal human esophageal epithelial-1 cell (Het-1A) and ESCC cell lines (Eca-109, KYSE-510, TE-1, KYSE-70, TE-11 and KYSE-150) were cultured in an incubator (Thermo Fisher Scientific, Waltham, MA, USA) that contained 5% CO2 and maintained at 37 °C. Culture medium contained penicillin-streptomycin, RPMI 1640 medium (Basal Media, China) and 10% fetal bovine serum (FBS; Biological Industries, Israel).

### Lentivirus-Mediated Small Hairpin RNA (LentishRNA) Against lnc-KIAA1244-2

Short-hairpin RNA (shRNA) against the target lnc-KIAA1244-2 and negative-control shRNA were constructed in a lentiviral vector (GeneChem, Shanghai, China). In accordance with the manufacturer’s instructions, shRNA was transfected into cells with lentiviral vector.

### CCK-8 assay

Cell Counting Kit-8 (CCK-8, Beyotime Biotechnology, Shanghai, China) was used to evaluate cell proliferation. Briefly, 96-well plates were seeded into 2000 cells, then incubated with 10 μl CCK-8 for 2 hours. The absorbance was measured at 450 nm by using a MultiSkan FC (Thermo Fisher Scientific Inc., Waltham, MA, USA.) at different time points.

### Cell cycle analysis

1 × 10^6^ Eca-109 cells were collected and fixed with ice-cold ethanol for 4 hour or more time. After the cells were washed with PBS, they were dyed with DNA staining solution (MultiSciences, Hangzhou, China) for 30 min in a dark place. Then, the cells were analyzed by flow cytometry.

### Western blotting analysis and antibodies

RIPA buffer (MultiSciences, Hangzhou, China) was used to extract the total protein. After the protein extracts were separated on 10% SDS-PAGE and electro transferred onto polyvinylidene difluoride (PVDF) membranes, the PVDF membranes were blocked with QuickBlock™ Blocking Buffer (Beyotime Biotechnology, Shanghai, China) for 20 min. Then, the membrane was immunostained with antibodies (anti-beta-actin antibody, Affinity; TNFAIP3 antibody, Novus) overnight at 4 °C. Subsequently, 1:5000 goat anti-rabbit or goat anti-mouse secondary antibodies were used for culturing 1 hour at room temperature. The bands were measured using a MiniChemi™ Imaging system and ImageJ software.

### Statistical analysis

Data analysis using SPSS software (version 19.0, Chicago, IL). The categorical data were analyzed by a chi-square test or Fisher exact tests, and displayed by cross tables. All experiments in the present study were repeated at least 3 times, and the data collected from 3 independent experiments are presented as the mean ± SD. The differences between the groups were assessed by Student’s t-test. All tests were two-sided. P-values < 0.05 were considered statistically significant.

### Ethics approval and consent to participate

Ethical approval for this study was obtained from the Institutional Ethics Committee of Hunan Cancer Hospital and with the 1964 Helsinki declaration and its later amendments.

## Supplementary information

supplementary data

## Data Availability

The datasets used during the current study are available from the corresponding author on reasonable request.
