# Author Correction: Genome-wide analyses of long non-coding RNA expression profiles and functional network analysis in esophageal squamous cell carcinoma

**DOI:** 10.1038/s41598-020-60113-3

**Published:** 2020-02-14

**Authors:** Junliang Ma, Yuhang Xiao, Bo Tian, Shaolin Chen, Baihua Zhang, Jie Wu, Zhining Wu, Xu Li, Jinming Tang, Desong Yang, Yong Zhou, Hui Wang, Min Su, Wenxiang Wang

**Affiliations:** 1 Department of the 2nd Department of Thoracic Surgery, Hunan Cancer Hospital and The Affiliated Cancer Hospital of Xiangya School of Medicine, Central South University, Changsha, Hunan 410013 P.R. China; 2 Hunan University of Medicine, Huaihua, Hunan 418000 P.R. China; 3 Hunan Key Laboratory of Translational Radiation Oncology, Hunan Cancer Hospital and The Affiliated Cancer Hospital of Xiangya School of Medicine, Central South University, Changsha, Hunan 410013 P.R. China; 4 Department of Pharmacy, Xiangya Hospital of Xiangya School of Medicine, Central South University, Changsha, Hunan 410001 P.R. China

Correction to: *Scientific Reports* 10.1038/s41598-019-45493-5, published online 24 June 2019

This Article contains errors.

The numbers of the differentially expressed lncRNAs and microRNAs were inadvertently swapped in the description of the Results. The numbers reported in the Abstract are correct.

As such, in the Results, in the section ‘Expression profiles of lncRNAs in ESCC’,

“Based on the microarray data, 3,052 lncRNAs and 2,366 mRNAs were identified to be differentially expression (FC ≥2.0 or ≤0.5, p ≤ 0.05).”

should read:

“Based on the microarray data, 2,366 lncRNAs and 3,052 mRNAs were identified to be differentially expression (FC ≥2.0 or ≤0.5, p ≤ 0.05).”

These errors do not affect the conclusions of the Article.

