# Supplementary material for: Genome-wide analyses of long non-coding RNA expression profiles and functional network analysis in esophageal squamous cell carcinoma
Source: Sci Rep. 2019 Jun 24;9:9162. doi: 10.1038/s41598-019-45493-5 (PMC6591223; doi:10.1038/s41598-019-45493-5)
Supplement: Supplementary file 1 — supplementary data [file 41598_2019_45493_MOESM1_ESM.pdf]

Supplementary Information

Genome-wide analyses of long non-coding RNA expression profiles and functional network analysis in esophageal squamous cell carcinoma

Junliang Ma[1,2], Yuhang Xiao[4], Bo Tian[1], Shaolin Chen[2], Baihua Zhang[1], Jie Wu[1], Zhining Wu[1], Xu Li[1], Jinming Tang[1], Desong Yang[1], Yong Zhou[1], Hui Wang[3], Min Su[1,3,*], Wenxiang Wang[1,3,*]

[1] Department of the 2nd Department of Thoracic Surgery, Hunan Cancer Hospital and The Affiliated Cancer Hospital of Xiangya School of Medicine, Central South University, Changsha, Hunan 410013, P.R. China;
[2] Hunan University of Medicine, Huaihua, Hunan 418000, P.R. China;
[3] Hunan Key Laboratory of Translational Radiation Oncology, Hunan Cancer Hospital and The Affiliated Cancer Hospital of Xiangya School of Medicine, Central South University, Changsha, Hunan 410013, P.R. China;
[4] Department of Pharmacy, Xiangya Hospital of Xiangya School of Medicine, Central South University, Changsha, Hunan 410001, P.R. China.

∗ Corresponding author:
Wenxiang Wang, No. 283 Tongzipo Road, Yuelu District, Changsha 410013, China (e-mail: hnchw11@163.com). Tel.: +86-731-8976-2111.
Min Su, No. 283 Tongzipo Road, Yuelu District, Changsha 410013, China (e-mail: sumin27@126.com). Tel.: +86-731-8865-1680.

Table S1 The primer sequences used in RT-PCR.

Gene symbol	Forward primer	Reverse primer
lnc-MMP1-2	CCATTGGATGGAGCTGCAAGG	GGACCACTGTCCTTTCTCCTAA
lnc-ABCA12-3	TGTGTGTCCTCCCATTTCCAGT	GCACCACTTTGCCACTCTCTTC
lnc-PTPN7-3	GTGCTTGGAGACCATCCCTC	CACACAGGGAGAGTTAGCGG
lnc-KIAA1244-2	CTGTTCAGCACGCTCAAGGA	GTCCCATTCATCATTCCAGTCC
lnc-SLC25A24-1	ATTACAGGTATCAGTTAAGAAAAC	CACCTTTAGCCGAGACAAAT
lnc-ARL4A-4	TGCTTCTTGCTGGATAGGGG	TCAGGGGTCAAGTCACAGTT
lnc-FBXL2-4	CGCGGGGCTGGGAGT	TGCTCACCAGCAAAGAATGC
lnc-SNRNP27-1	CCCTCCCTCTCTTGTCGAGC	TCAGTTCCCTCAACCCAAACA
TNFAIP3	TCCTCAGGCTTTGTATTTGAGC	TGTGTATCGGTGCATGGTTTTA
GAPDH	ACAGCCTCAAGATCATCAGC	GGTCATGAGTCCTTCCACGAT

Table S2 The top 30 co-expressed genes of lnc-MMP10-3.

Gene Symbol	Correlation	*p*-value	Gene Symbol	Correlation	*p*-value
HMX1	-0.884	0.00069	ISY1	0.749	0.013
NOSTRIN	-0.816	0.004	EFNA5	-0.766	0.0098
HLA-DRA	0.727	0.017	NRG2	-0.879	0.00081
IQGAP3	0.804	0.005	ELOVL6	-0.953	0.000019
C1orf131	0.818	0.0038	IVNS1ABP	0.836	0.0026
EHD3	-0.969	0.0000037	CMPK1	-0.935	0.000071
LRRFIP2	-0.916	0.00019	ATP6V1C2	-0.835	0.0027
GCNT4	-0.88	0.00078	ADCY3	0.798	0.0057
MCM6	0.887	0.00061	FHIT	-0.767	0.0096
PIK3R4	0.908	0.00028	ADORA3	0.851	0.0018
ADAM17	0.766	0.0098	NCAPH	0.866	0.0012
CSMD2	0.756	0.011	ANKUB1	0.702	0.024
LOC101927282	0.914	0.00022	RCAN2	-0.729	0.017
LOC101927933	0.847	0.002	VNN1	0.801	0.0054
CTC-484P3.3	0.762	0.01	SPAG17	-0.715	0.02

Table S3 The top 30 GO analysis enrichment terms of lnc-MMP10-3.

Enrichment term	Description	ListHits	*p*-value
GO:0000062	fatty-acyl-CoA binding	4	0.039092616
GO:0000082	G1/S transition of mitotic cell cycle	19	0.014577171
GO:0000139	Golgi membrane	52	0.000911013
GO:0000165	MAPK cascade	11	0.037684335
GO:0000213	tRNA-intron endonuclease activity	2	0.034573763
GO:0000278	mitotic cell cycle	50	0.000067654
GO:0000774	adenyl-nucleotide exchange factor activity	2	0.049732165
GO:0000775	chromosome, centromeric region	14	0.000057233
GO:0000776	kinetochore	12	0.008659268
GO:0000777	condensed chromosome kinetochore	12	0.004920571
GO:0000778	condensed nuclear chromosome kinetochore	2	0.049732165
GO:0000780	condensed nuclear chromosome, centromeric region	3	0.027733769
GO:0000786	nucleosome	11	0.004476649
GO:0000922	spindle pole	12	0.031407415
GO:0000940	condensed chromosome outer kinetochore	3	0.043791450
GO:0001106	RNA polymerase II transcription corepressor activity	5	0.036977788
GO:0001501	skeletal system development	16	0.022042227
GO:0001533	cornified envelope	5	0.028252085
GO:0001660	fever generation	3	0.004256957
GO:0001736	establishment of planar polarity	4	0.009259407
GO:0001740	Barr body	2	0.049732165
GO:0001772	immunological synapse	6	0.020345658
GO:0001812	positive regulation of type I hypersensitivity	2	0.021637261
GO:0001824	blastocyst development	4	0.039092616
GO:0001960	negative regulation of cytokine-mediated signaling pathway	3	0.035290613
GO:0001968	fibronectin binding	6	0.013315104
GO:0002020	protease binding	13	0.002904858
GO:0002084	protein depalmitoylation	2	0.049732165
GO:0002224	toll-like receptor signaling pathway	14	0.028022516
GO:0002238	response to molecule of fungal origin	2	0.021637261

Table S4 KEGG pathway analysis of lnc-MMP10-3 co-expressed genes.

Enrichment term	Description	List Hits	PopHit	*p*-value
path:hsa00062	Fatty acid elongation	6	23	0.014734356
path:hsa00120	Primary bile acid biosynthesis	5	17	0.017184869
path:hsa00280	Valine, leucine and isoleucine degradation	11	44	0.00145349
path:hsa00480	Glutathione metabolism	9	51	0.025034732
path:hsa00520	Amino sugar and nucleotide sugar metabolism	9	47	0.016485121
path:hsa00640	Propanoate metabolism	6	28	0.030904819
path:hsa00790	Folate biosynthesis	4	14	0.034967181
path:hsa01100	Metabolic pathways	110	1213	0.037625419
path:hsa01212	Fatty acid metabolism	8	47	0.039523252
path:hsa03320	PPAR signaling pathway	11	69	0.02556494
path:hsa04145	Phagosome	22	155	0.007163013
path:hsa04380	Osteoclast differentiation	17	131	0.033596691
path:hsa04512	ECM-receptor interaction	15	87	0.005452861
path:hsa04620	Toll-like receptor signaling pathway	14	106	0.045395416
path:hsa04660	T cell receptor signaling pathway	15	104	0.021313128
path:hsa04710	Circadian rhythm	6	31	0.044680951
path:hsa04940	Type I diabetes mellitus	10	45	0.004889357
path:hsa05020	Prion diseases	8	36	0.01136858
path:hsa05100	Bacterial invasion of epithelial cells	11	76	0.043846974
path:hsa05133	Pertussis	12	75	0.019765979
path:hsa05140	Leishmaniasis	14	74	0.003482285
path:hsa05322	Systemic lupus erythematosus	24	136	0.000372996
path:hsa05332	Graft-versus-host disease	8	43	0.026449731

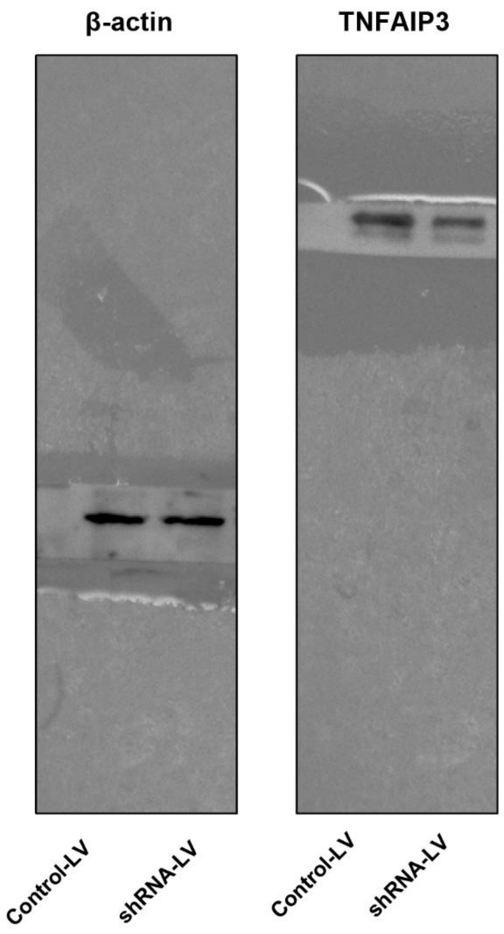

Figure S1, related to Figure 6. The full-length blots/gels.
